# Biomarkers of UVB radiation-related senescent fibroblasts

**DOI:** 10.1038/s41598-023-51058-4

**Published:** 2024-01-09

**Authors:** Mingyue Qiang, Zijia Dai

**Affiliations:** Department of Dermatology, The Second Affiliated Hospital of Nanjing Medical University, Nanjing, 210003 China

**Keywords:** Computational biology and bioinformatics, Biomarkers, Skin diseases

## Abstract

Excessive exposure to ultraviolet (UV) light is known to induce photoaging in the skin, necessitating the development of effective anti-photoaging strategies to mitigate the adverse effects of UV radiation. Understanding the biofunctional characteristics of diverse skin cell types and unraveling the molecular modifications implicated in the aging process are pivotal in comprehending the intricacies of photoaging in human skin. Such insights are essential for paving the way for innovative interventions to counteract the deleterious impact of UV radiation on the skin. The single-cell RNA sequencing data of UVB-irradiated and normal control mouse skin in GSE173385 were downloaded from the Gene Expression Omniniub (GEO) database. First, cell types were identified using Seurat for normalization, dimensionality reduction and clustering. Next, Gene Ontology (GO) and Kyoto Encyclopedia of Genes and Genomes (KEGG) functional enrichment analysis were executed on these cell subpopulations. Using FindAllMarkers in the Seurat package to identify differential gene expression and Monocle2 cell trajectory analysis, we screened out hub genes related to the development trajectory of senescent fibroblasts during photoaging, and then combined it with 307 aging-related genes collected in the HAGR library, we finally identified two biomarkers. The efficiency of biomarkers in diagnosing UV radiation photoaging was also evaluated in the dataset. Concurrently, the immune infiltration of identified biomarkers under UV radiation has also been further explored. Moreover, we employed the Enrichr platform to conduct a comprehensive screening of drug molecules associated with the identified biomarkers. Our comprehensive analysis, employing Seurat for normalization, dimensionality reduction, and clustering, successfully identified ten distinct cell types within the samples. Then GO functional enrichment analysis showed that senescent fibroblasts are mainly involved in the regulation of immune effector processes such as cytokine-mediated signaling pathways, regulation of epithelial cell proliferation and intercellular adhesion. Afterwards, KEGG analysis determined the main biological pathways are: IL-17 signaling pathway, Cytokine–cytokine receptor interaction, Metabolism of xenobiotics by cytochrome P450. After differential gene expression and Monocle2 cell trajectory analysis, we matched the obtained hub genes with the aging-related genes collected in the HAGR library, and finally screened out two relevant biomarkers: Apoe and Gdf15 which are related to the development trajectory of senescent fibroblasts during photoaging. Meanwhile, the immune infiltration further implied that the expression of these two biomarkers was significantly correlated with immune cells. In addition, the Enrichr platform was used to screen the drug molecules related to these biomarkers. This strategic approach aimed to pinpoint effective molecular targets for the prevention and treatment of photoaging. Our investigation has effectively characterized biomarkers associated with fibroblast senescence during photoaging at the single-cell level, We have validated their correlation with cellular immune inflammation and identified potential drug targets through the utilization of the Enrichr platform. This foundational research establishes a robust basis for the development of therapeutic interventions targeting skin diseases resulting from photoaging.

## Introduction

The skin functions as a crucial barrier against environmental threats, and ultraviolet B (UVB) radiation, a constituent of sunlight, has been implicated in the disruption of the skin barrier^1^. Reduction in stratospheric ozone results in increased UVB radiation, penetrating the epidermis and reaching the upper dermis. This process promotes the generation of reactive oxygen species (ROS), triggers the inflammatory response, and facilitates the accumulation of DNA damage. Consequently, the skin experiences increased stress and becomes more susceptible to the development of cancer^2,3^.

During the aging process, skin cells undergo intricate changes, often accompanied by alterations in gene expression and cellular transitions^4,5^. Within the dermis, fibroblasts, comprising the majority of the cell population, continuously undergo damage accumulation and (mal-)adaptation, either directly or through interactions with other cells, representing a pivotal element in skin homeostasis and aging that should not be underestimated^6^. Cell-autonomous and exogenous stressors can induce permanent senescence in resting fibroblasts, leading to telomere shortening^7^, mitochondrial dysfunction^8^, DNA damage^9^, and activation of the DNA damage response signaling pathway. These processes ultimately culminate in induced cellular senescence^10^. Understanding fibroblast senescence is crucial for advancing our comprehension of the mechanisms underlying skin aging.

In light of the rapid advancements in machine learning, this method has been extensively employed in investigating the pathophysiology of various immunological and inflammatory-related diseases. In this study, we conducted a comprehensive analysis utilizing single-cell RNA sequencing (scRNA-seq) analysis on both normal skin and skin post-UVB radiation exposure. Through our scRNA-seq analysis, we identified ten distinct subpopulations of skin cells, each characterized by unique gene expression profiles. Subsequent functional annotation studies were undertaken for each identified cell subpopulation. Integration of differential expression analysis and cell trajectory analysis was employed to systematically identify potential biomarkers associated with fibroblast senescence induced by UVB radiation. This synergistic approach was adopted to elucidate the underlying mechanism of cell damage resulting from UVB exposure.

## Materials and methods

### Sepsis data collection and acquisition of AGs

The single-cell transcriptomics dataset GSE173385 acquired from the GEO database (https://www.ncbi.nlm.nih.gov/geo/), was based on the GPL24247 platform. This dataset encompassd gene expression profiles obtained from the skin of normal and UV-irradiated mice. A total of 307 Ageing-Related Genes (AGs) were collected in total from HAGR (The Ageing Gene database) (http://genomics.senescence.info/genes/)^11^.

### Single-cell RNA-seq studies

Single skin cells from both untreated and UVB-irradiated mice were subjected to normalization, dimensionality reduction, and clustering. The process was carried out by utilizing the Seurat package (version 2.3.4; https://satijalab.org/seurat/install.html)^12^. The LogNormalize method method was employed, and a scale factor that was specified as the average of column sums across the expression matrix.

### Differential gene expression analysis

The FindAllMarkers function in the Seurat package (settings: min.pct = 0.25, thresh.use = 0.25) was used to compare each cluster to all the others to find DEGs; only those with |'avg logFC'|> 0.25 and ‘*p*_val_adj’ < 0.05 were determined to be DEGs (differentially expressed genes) related with senescent fibroblasts.

### Creating trajectories for a single cell

In order to determine the transitions between cell states, a single cell trajectory analysis was performed using the Monocle2 program (version 2.8.0). We were able to sort the cells into a pseudo-time order by using differentially expressed genes in fibroblasts and senescent fibroblasts that were discovered using Seurat. During the initial "orderCells" phase, the ductal state provided us with information on the beginning of the pseudotime. After that, we gave the ductal cells that had aberrant gene expression profiles as the root state option to the second instance of the 'orderCells' command that we issued. The 'DDRTree' algorithm was used to minimize the dimensions, and the 'plot cell trajectory' visualization function was utilized so that the least spanning tree could be plotted on the cells. The "differentialGeneTest" function in Monocle2 was used to determine the differentially expressed genes during the course of the Pseudo-time from ductal cells with aberrant gene expression patterns to malignant ductal cells transition. The q value for this function was set to be less than 1e-4.

### Fibroblasts senescent-related hub genes

We further intersected DEGs and genes potentially associated with fibroblast senescent (FSGs) to obtain common genes. These genes were considered hub genes. The "Venndiagram" program was used to create a Venn diagram depicting the total number of DEG-FSGs. The pROC program was utilized to determine the area under the receiver operating characteristic curve (AUC) for the hub gene^13^.

### Functional and pathway enrichment analyses

Gene ontology is a bioinformatics tool that allows the classification of genes and genomic products into different groups based on common properties^14^. KEGG^15^ is a database involving information on genomes, biological pathways, diseases, and chemicals. In this study, we used the clusterProfiler package to perform GO and KEGG pathway enrichment analysis of common genes *P* < 0.05 indicated statistical significance for enrichment. A computer tool, GSEA compares two biological states to see if there are statistically significant differences in an a priori determined genome. GSEA enrichment was performed using the clusterProfiler package (3.14.3) in R to determine the overall gene enrichment differences^16^. We downloaded the h.all.v7.0.symbols.gmt from the Molecular Signatures Database (http://www.broad.mit.edu/gsea/msigdb/). A false discovery rate (FDR) < 25% and nominal *P* value < 5% were set as the cut-off criteria.

### Analysis of the relationship between genes and immune cells

We used the CIBERSORT deconvolution algorithm to characterize cell composition^17^. For this study, we utilized cibersort.R (available for free at http://cibersort.stanford.edu/) to analyze the abundance of 22 invasive immune cells in skin cells exposed to UVB radiation. The LM22 leukocyte signature matrix is a feature matrix that is used by cibersort. There are 547 genes for which expression data and matrices are stored in LM22.

### Analysis of the hub gene's expression by GSE41078 and GSE54413 datasets

To examine hub gene expression, the GEO datasets GSE41078 and GSE54413 were imported. The dataset GSE41078 encompasses 10 normal samples and 10 UVB-treated skin samples, while the GSE54413 dataset comprises 15 normal samples and 15 UVB-treated skin samples, as outlined in Table 1. A comparison of untreated and UVB-treated skin samples was conducted by Student's t-test in order to locate the differentially expressed genes (DEGs).

**Table 1:** Characteristics of validation sets in this study.

GSE series	Platform	Total	UVB-treated	Control
GSE41078	GPL571	20	10	10
GSE54413	GPL6480	30	15	15

### The identification of potential medication candidates

The identification of therapeutic compounds has emerged as the primary focus of this area of research in recent years. A senescence-related therapeutic molecule is created with the use of the Drug Signatures database (DSigDB), which has 22,527 different gene sets. Enrichr may be accessed at the following URL: https://amp.pharm.mssm.edu/Enrichr/. This platform is used to gain access to the DSigDB database. Enrichr is mostly utilized as a platform for enrichment analysis since it demonstrates a large number of visualization features on collective functions for the genes that are supplied as input^18^.

## Results

### Diverse cell types in skin cells after UVB irradiation delineated by single cell transcriptomic analysis

**Figure 1:**
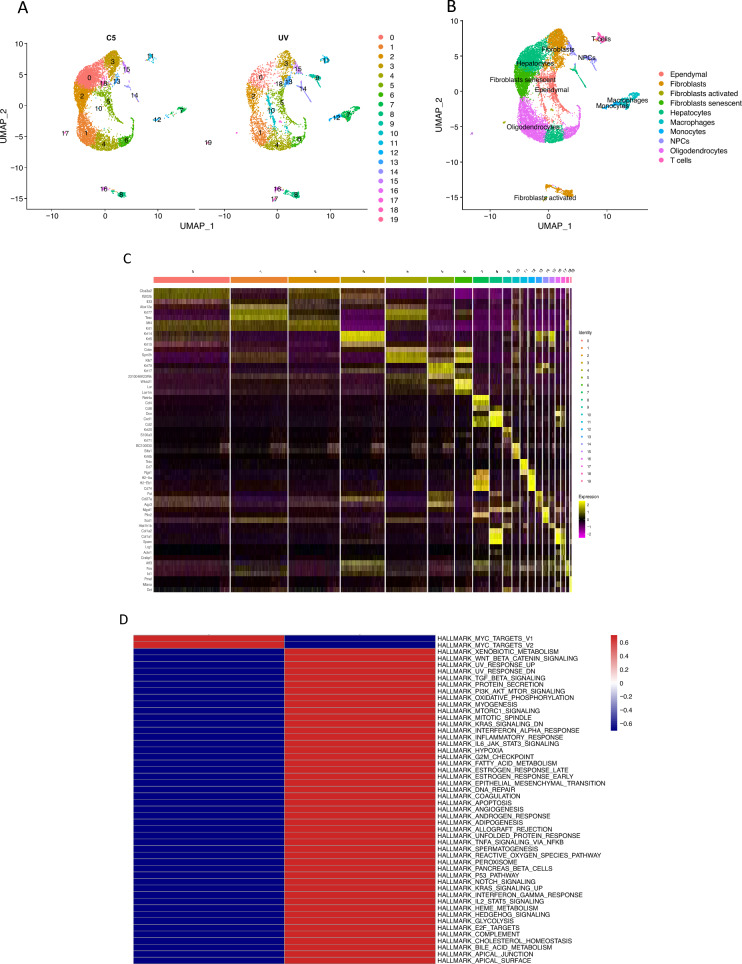
sc-RNA Analysis in normal and UVB-irradiated skin cells. (**A**) Dimensionality reduction and clustering of normal (C5) and UVB-irradiated (UV) skin cells. Cluster-based coloring of all cells in a UMAP diagram. (**B**) UMAP plot of normal and UV-irradiated skin cells, colored by 10 major cell types. The right side of each cluster displays the anatomical location that has been assigned to it. (**C**) A heatmap displays the canonical cell-type markers of the 10 major cell types. (**D**) GSEA analysis of hall marks in UVB-irradiated skin cells.

We procured single-cell RNA-seq profiles from both untreated and UVB-irradiated cells. Initial steps involved clustering and dimensionality reduction of gene expression profiles extracted from the skin of untreated mice and those exposed to UV radiation. Following rigorous quality assessment, single-cell transcriptome data were compiled from 5028 cells in UVB-irradiated samples and 8316 cells in control samples. Ten main cell types were discerned utilizing canonical cell markers: Ependymal, Fibroblasts, Fibroblasts activated, Fibroblasts senescent, Hepatocytes, Macrophages, Monocytes, NPCs, Oligodendrocytes, T cells (Fig. 1A–C). Functional enrichment analyses unveiled that genes in UVB-exposed cells were exhibited pronounced enrichment in pathways associated with aging, encompassing DNA repair, inflammatory response, apoptosis, and unfolded protein response, among others (Fig. 1D).

### Differential gene expression (DGE) analysis and functional enrichment analysis of UVB-irradiated skin cells subtype

**Figure 2:**
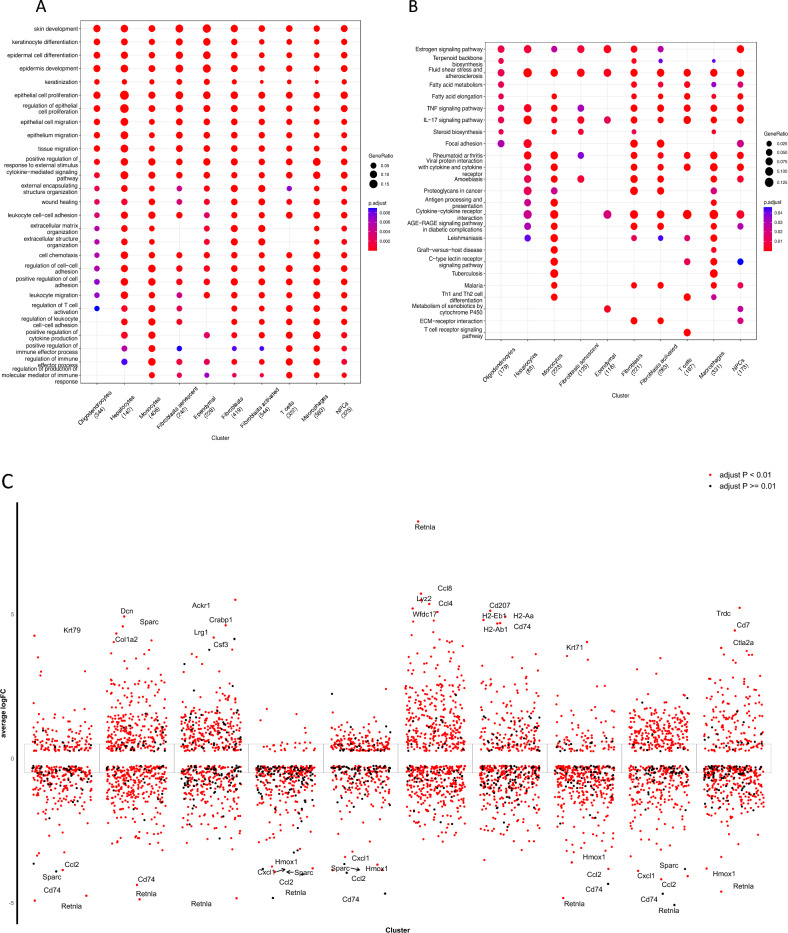
Functional enrichment analysis of each Cell Types in skin UVB irradiation. (**A**) GO terms of each cluster of DGEs in UVB-irradiated skin cells. (**B**) Bubble map of the KEGG enrichment analysis of each cluster of DEGs in UVB-irradiated skin cells. The X-axis displays the various cell types while the Y-axis displays the various classifications. The size of the circle corresponds to the total amount of enriched genes within a subset. Different qualities are represented by each circle hue. (**C**) Analysis of difference in gene expression exposed to UVB showed up- and down-regulated genes in all 10 clusters. An adjusted *p* value < 0.01 is indicated in red, while an adjusted *p* value >  = 0.01 is indicated in black.

Through the preceding analysis, we identified 10 distinct cell types in UVB-irradiated skin cells. Our investigation into Gene Ontology (GO) enrichment delved into the expression patterns of significant pathways within each of these cell types. Notably, genes associated with Senescent Fibroblasts exhibited notable associations with the regulation of immune effector processes, cytokine-mediated signaling pathways, epithelial cell proliferation, and the regulation of cell–cell adhesion (Fig. 2A). Subsequently, a Kyoto Encyclopedia of Genes and Genomes (KEGG) analysis was undertaken to unveil the primary biological pathways preferentially activated across various cell types. Within the Fibroblasts senescent subgroup, seven KEGG terms were identified as enriched (Fig. 2B). Differential gene expression analysis (DEGs, |avg_logFC|> 0.25 and *p*_val_adj < 0.05) was then executed between clusters in UVB-irradiated cells (Fig. 2C). Ultimately, we identified 293 differentially expressed genes related to fibroblast senescence.

### Detailed analysis of fibroblast senescence trajectory and identification of key genes

**Figure 3:**
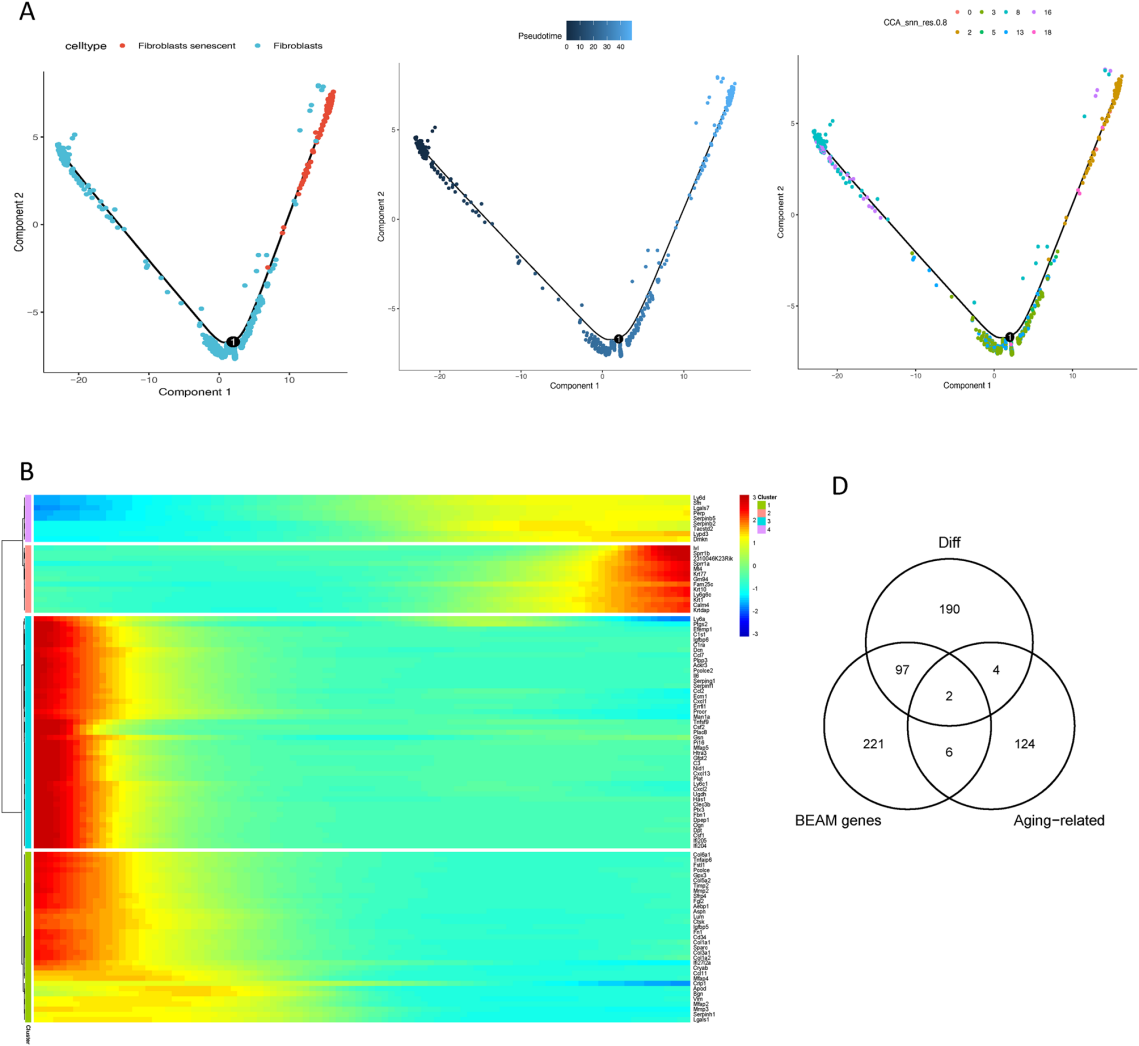

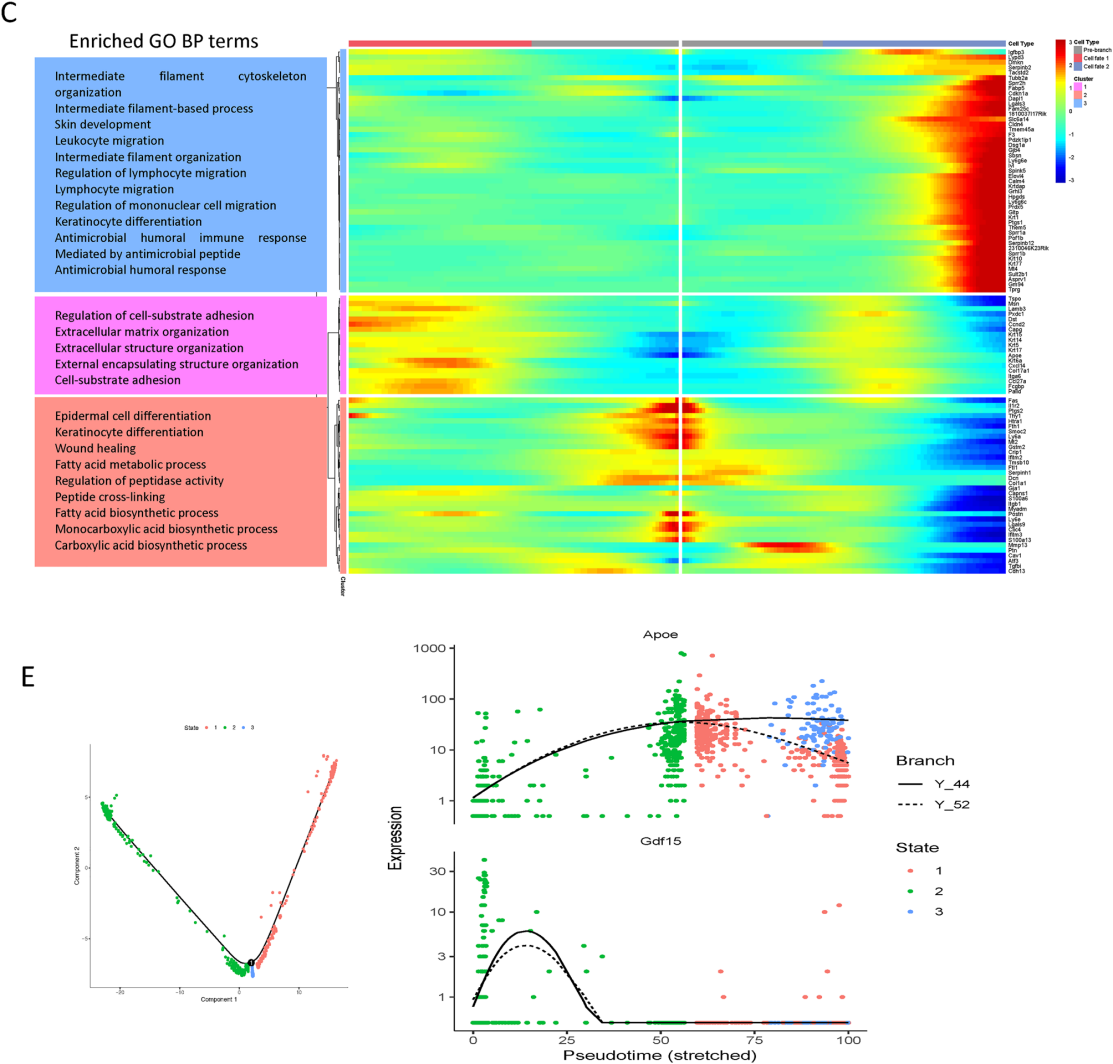
Gene expression patterns differ as fibroblasts develop. (**A**) Trajectory diagram of Monocle after sorting of single cells. The horizontal coordinates indicate the two major components, each spot on the plot represents a cell, and the numbers in the black circles are the nodes in the trajectory analysis that define the various cell states. (The different colors in the left panel represent the different cell types, the middle panel is colored from dark to light in the proposed time order, and the different colors in the right panel represent the different cell Clusters) (**B**) Heat map of the top 100 genes that were expressed differently along pseudo-time (rows) and pseudo-time (columns), grouped hierarchically into four shapes. The genes are shown in the rows, and the probable time points are shown in the columns; the colors indicate the average expression levels of those genes at that moment; and the expression levels decrease from red to blue as time progresses. (**C**) The heat map shows the first 100 genes obtained from the analysis of the specified branch points in A. The top is the node minutes in the proposed time sequence, cell fate1 represents fibroblasts, and cell fate2 represents senescent fibroblasts. The left side of the heat map shows the GO function analysis for each cluster. (**D**) Genes linked with senescence and those differently expressed both at the pseudo-time nodes of the trajectory analysis and in senescent fibroblasts overlap in this Venn diagram. (**E**) Scatter plot of the expression pattern of Apoe, Gdf15 along the pseudo-time.

The aging process of cells is fundamental to skin aging, with cellular aging serving as the underlying cause for all manifestations of aging. To investigate the lineage of senescent cells, trajectory analysis was conducted on fibroblasts and senescent fibroblasts to delineate their developmental pathways within skin cells using Monocle2. As revealed by Monocle, the data suggested the potential transition of fibroblasts to senescent fibroblasts (Fig. 3A). The 'differentialGeneTest' function identified differentially expressed genes based on proposed time values and visualized the clustering of these genes, enabling the observation of changes in individual gene modules during the specified time (Fig. 3B). Branching points in the proposed temporal analysis results indicated cells undergoing fate differentiation. The BEAM method in Monocle was employed to analyze cellular data and nodes post-temporal sorting, leading to the identification of differentially expressed genes associated with branching. Subsequently, functional analysis was performed on these genes. Functional enrichment analysis results revealed that genes in cluster 3 were predominantly enriched in the organization of intermediate filaments in the cytoskeleton, skin development, and immune cell migration. Cluster 1 exhibited abundance in extracellular structure organization and regulation of cell-substrate adhesion. Additionally, functional enrichment analysis of cluster 2 indicated that genes in this cluster were primarily associated with skin cell differentiation, including epidermal and keratinocyte differentiation, wound healing, fatty acid biosynthesis, and metabolism (Fig. 3C).

Subsequently, the intersection of differentially expressed genes in senescent fibroblasts, genes with dynamic expression changes from the BEAM analysis, and aging-related genes from HAGR was examined. Finally, two key genes, Gdf15 and Apoe, were identified and screened (Fig. 3D). The genetic evolution of these key genes was depicted from the beginning to the end of the hypothesized timeline in Fig. 3E.

### ROC curve of the key genes in UVB-treated samples

**Figure 4:**
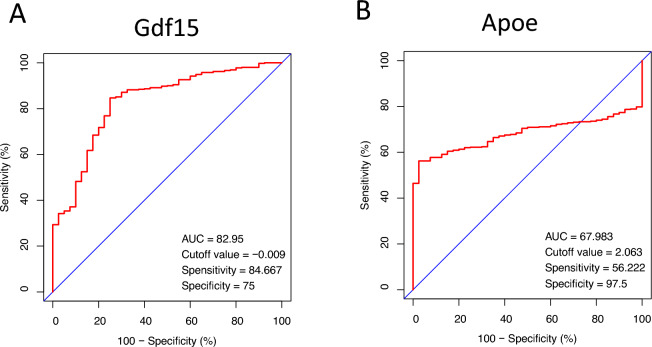
ROC curve of 2 key genes (**A**) Gdf15, (**B**) Apoe in UVB-treated samples. AUC area under the ROC curve.

We generated Receiver Operating Characteristic (ROC) curves and calculated the associated Area Under the Curve (AUC) for the gene expression levels in the UVB-treated datasets, with the objective of validating the diagnostic significance of the two primary genes identified in the preceding analysis. The diagnostic efficacy of these tests is effectively summarized by their AUC, a metric that amalgamates sensitivity and specificity. As illustrated in Fig. 4, Gdf15 (AUC: 0.8295) and Apoe (AUC: 0.6798) demonstrated superior diagnostic value in the UVB-treated samples.

### Validation of ROC curve and hub genes expression

**Figure 5:**
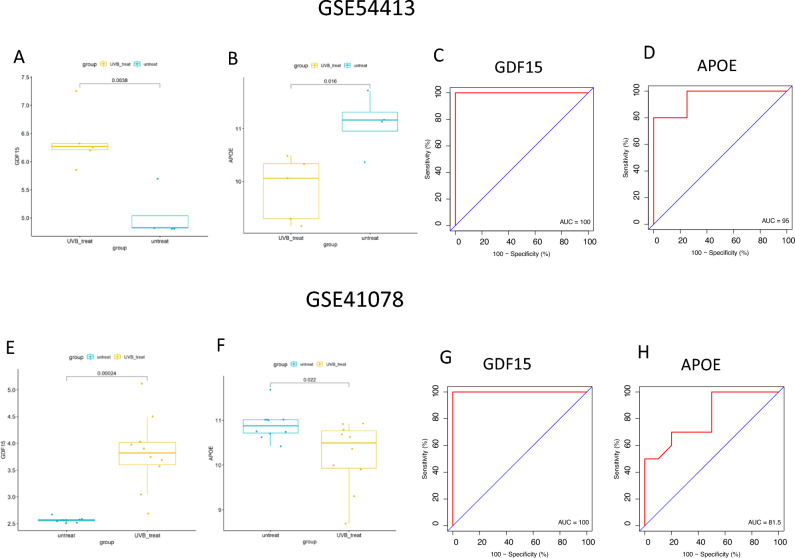
The mRNA expression and ROC diagnostic curve of the hub genes was determined from the GSE54413 dataset: (**A**, **C**) Gdf15; (**B**, **D**) Apoe; and form the GSE41078 dataset: (**E**, **G**) Gdf15; (**F**, **H**) Apoe.

To verify the expression levels of the two pivotal genes, the GSE41078 and GSE54413 datasets were utilized as validation sets. The expression of Gdf15 was increased in UVB-irradiated skin, while Apoe expression demonstrated a conversely decreased trend (Fig. 5A, B, E and F). Subsequently, we validated the diagnostic values using ROC analysis with the validation dataset, and both Gdf15 and Apoe demonstrated ROC values exceeding 80% (Fig. 5C, D, G and H). These results suggest that our identified key genes, Gdf15 and Apoe, possess high diagnostic value. Consequently, we propose that Gdf15 and Apoe may serve as potential biomarkers for UVB-induced damage based on our current sample set.

### Immune landscape of UVB-treated groups

**Figure 6:**
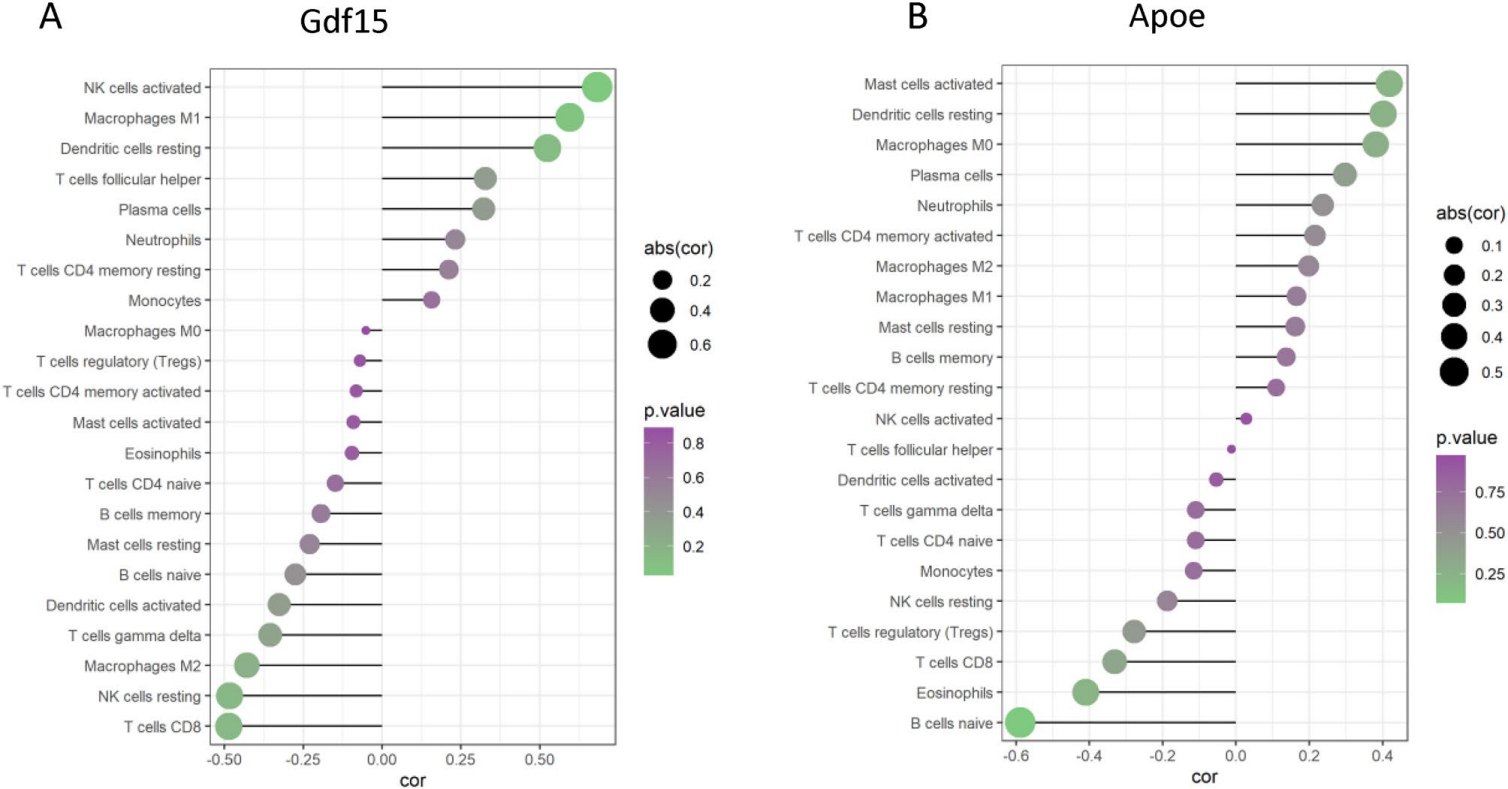
The relationship between gene expression and the ratio of immune cell infiltration. (**A**) Gdf15's lollipop chart, (**B**) Lollipop plot of Apoe. The ball’s size shows the correlation strength as determined by Spearman analysis.

Furthermore, in our exploration of immune cell infiltration in UVB-irradiated mouse skin cells, we evaluated the associations between the dataset and the immunological microenvironment using the CIBERSORT approach. Subsequently, the correlation between key genes and immune cells was examined. The expression of Gdf15 (Fig. 6A) exhibited a significant positive correlation with the infiltration ratio of activated NK cells, with a correlation coefficient of 0.68 (*P* = 0.02). Conversely, Apoe (Fig. 6B) displayed a notable negative correlation with the infiltration rate of naive B cells (cor = − 0.59, *P* = 0.04).

### The identification of potential medication candidates

The Enrichr platform was employed to identify potential therapeutic compounds for the previously evaluated hub genes, leveraging data from the DSigDB database. Candidate medications were selected based on both the *P*-value and the corrected *P*-value. As depicted in Table 2 COBALT CTD 00005696 and 17-Hydroxyandrostan-3-one CTD 00006776 are two medication molecules that interact with the majority of genes in UVB-irradiated skin cells. These findings were extracted from an analysis of the data presented in the Table2. Notably, these signature pharmaceuticals were found to be associated with a high frequency of Differentially Expressed Genes (DEGs), suggesting their representation as commonly encountered substances in the context of UVB irradiation.

**Table 2:** Suggested top drug compounds.

Name of drugs	*p*-value	Adjusted *p*-value	Genes
COBALT CTD 00005696	0.00007526	0.01582	Apoe, Gdf15
17-Hydroxyandrostan-3-one CTD 00006776	0.00008789	0.01582	Apoe, Gdf15
Alsterpaullone MCF7 UP	0.0001632	0.01654	Apoe, Gdf15
Rosiglitazone CTD 00003139	0.0004233	0.01654	Apoe, Gdf15
Aspirin CTD 00,005,447	0.0007882	0.01654	Apoe, Gdf15
NICKEL SULFATE CTD 00001417	0.0008309	0.01654	Apoe, Gdf15
Troglitazone CTD 00002415	0.001061	0.01654	Apoe, Gdf15
Vitinoin CTD 00007069	0.001523	0.01654	Apoe, Gdf15
Tamoxifen CTD 00006827	0.001610	0.01654	Apoe, Gdf15
Sulfaguanidine PC3 UP	0.001634	0.01654	Apoe, Gdf15

## Discussion

Cellular senescence signifies a state of growth arrest and compromised function observed in mammalian cells in response to injury or stress, with implications for contributing to the aging process^19–21^. Nevertheless, the intricate mechanism by underlying its influence remains poorly elucidated. In addition to impeding the body's regenerative capacity, senescent cells may pose harm by releasing substances that disrupt tissue homeostasis^22,23^. Through the secretion of cytokines, extracellular matrix, and degrading enzymes, senescent fibroblasts can exert a profound impact on adjacent epithelial cells^24,25^. Consequently, this influence may potentiate the proliferation of epithelial cells harboring mutations, thereby contributing to tumorigenesis^26^.

An elevation in the ultraviolet B radiation reaching the Earth's surface has ensued as a direct consequence of ozone layer thinning in the stratosphere. This escalation presents notable hazard to cutaneous integrity, as it heightens the prospect of enduring damage with potential long-term ramifications, notably photoaging and photocarcinogenesis. Previous investigations have delineated that UVB-induced photoaging is associated with anomalous alterations in primary skin fibroblasts and keratinocytes^27,28^. A meticulous examination of fibroblast modifications under UVB radiation serves to enhance our comprehension of the mechanistic nuances intrinsic to photoaging.

In our investigation, we characterized ten cell types in UVB-irradiated skin cells, and screened 293 key genes that differentially expressed in senescent fibroblasts. GO enrichment analysis indicated that senescent fibroblasts were mainly associated with skin development, keratinocyte differentiation, cytokine–mediated signaling pathway. KEGG pathway annotation analysis revealed that senescent fibroblasts were mainly involved in TNF signaling pathway, IL-17 signaling pathway. Our findings align with existing literature on UV radiation-induced inflammation and immunosuppression, known to hasten the aging process of the skin. The observed associations underscore the relevance of these pathways in elucidating the molecular mechanisms underlying the impact of UVB radiation on skin aging^29^.

As revealed by Monocle, fibroblasts differentiate into two main branches, fibroblasts and senescent fibroblasts. Subsequently, we meticulously screened 400 related genes along the developmental pathway from fibroblasts to senescent fibroblasts. Through the strategic intersection of these genes with the previously identified differentially expressed genes and those associated with senescence (Obtained from The Ageing Gene database), we selected two hub genes: Apoe, Gdf15.

Apolipoprotein E (Apoe) is a pivotal protein with essential functions in mammalian physiology, primarily concerning lipid binding. Its predominant presence is noted in very-low-density lipoproteins (VLDL), celiac particles (CM) and their remains^30^. Apolipoproteins play a crucial role in lipoprotein metabolism, facilitating the transport of triglycerides and cholesterol between different tissues^31^. Notably, Apoe deficiency in mice (apoE-/-) has been associated with aging-related manifestations, including skin thinning and a reduction in collagen density, suggesting a role in maintaining skin integrity^32^. Our study aligns with these findings, revealing abnormal expression of the APOE gene in response to aging induced by light radiation. However, further experimental validation is needed to delineate the precise mechanism by which APOE contributes to UVB-induced aging. Targeting APOE-associated pathways offers a promising avenue for modulating inflammatory states and lipid metabolism, with the potential to mitigate the aging symptoms triggered by light radiation. This underscores the importance of identifying therapeutic targets for efficacious intervention in skin aging processes initiated by environmental factors such as light radiation.

Gdf15, short for growth/differentiation factor 15, belongs to the class of proteins known as the transforming growth factor beta proteins, has emerged as a key player in senescent fibroblasts. Previous studies have reported that GDF15 from senescent fibroblasts can stimulate skin pigmentation under UVB exposure, suggesting its potential involvement in age-associated pigmentation changes^33^. At the same time, ultraviolet B radiation (UVB) has been shown to activate various inflammation-related pathways, leading to the secretion of cytokines by human keratinocytes. Notably, GDF-15 is among the cytokines whose expression is heightened during both inflammation and aging^34^. Consistent with existing literature, our results underscore a role for GDF15 in light radiation-induced senescence. However, to elucidate the underlying mechanisms, further experiments are imperative. Verification of these mechanisms will enhance our understanding of the intricate interplay between GDF15 and the molecular pathways implicated in light radiation-induced skin aging.

## Conclusion

UVB radiation is implicated in various skin-related issues, encompassing photoaging, skin inflammation, and the potential development of skin cancer. In our study, we discerned pivotal genes and molecular pathways integral to the fibroblast senescence triggered by UVB radiation, through the analysis of single-cell analysis post-UVB exposure. Additionally, we employed these insights to predict potential drugs targets based on identified key genes, aiming to modulate skin immune inflammation as a strategic approach for addressing skin diseases arising from photoaging. In general, our findings offer novel insights into the pathological mechanisms and therapeutic strategies for managing photoaging.

## Data Availability

The datasets analysed during the current study are available in the [GEO] repository. [https://www.ncbi.nlm.nih.gov/geo/query/acc.cgi?acc=GSE173385]. Users can download relevant data for free for research and publish relevant articles.
